# On the correlation between outcome indicators and the structure and process indicators used to proxy them in public health care reporting

**DOI:** 10.1007/s10198-021-01333-w

**Published:** 2021-06-30

**Authors:** Benjamin H. Salampessy, France R. M. Portrait, Eric van der Hijden, Ab Klink, Xander Koolman

**Affiliations:** 1 Department of Health Sciences, Faculty of Science, Vrije Universiteit Amsterdam, De Boelelaan 1085, 1081 HV Amsterdam, The Netherlands; 2 Department of Political Science and Public Administration, Faculty of Social Sciences, Vrije Universiteit Amsterdam, De Boelelaan 1085, 1081 HV Amsterdam, The Netherlands

**Keywords:** Quality measurement, Profiling providers, Publicly reported quality indicators, Hospital standardized mortality ratios, Health policy, I11 Analysis of health care markets, I18 Government policy, Regulation, Public health

## Abstract

Hospital quality indicators provide valuable insights for quality improvement, empower patients to choose providers, and have become a cornerstone of value-based payment. As outcome indicators are cumbersome and expensive to measure, many health systems have relied on proxy indicators, such as structure and process indicators. In this paper, we assess the extent to which publicly reported structure and process indicators are correlated with outcome indicators, to determine if these provide useful signals to inform the public about the outcomes. Quality indicators for three conditions (breast and colorectal cancer, and hip replacement surgery) for Dutch hospitals (2011–2018) were collected. Structure and process indicators were compared to condition-specific outcome indicators and in-hospital mortality ratios in a between-hospital comparison (cross-sectional and between-effects models) and in within-hospital comparison (fixed-effects models). Systematic association could not be observed for any of the models. Both positive and negative signs were observed where negative associations were to be expected. Despite sufficient statistical power, the share of significant correlations was small [mean share: 13.2% (cross-sectional); 26.3% (between-effects); 13.2% (fixed-effects)]. These findings persisted in stratified analyses by type of hospital and in models using a multivariate approach. We conclude that, in the context of compulsory public reporting, structure and process indicators are not correlated with outcome indicators, neither in between-hospital comparisons nor in within-hospital comparisons. While structure and process indicators remain valuable for internal quality improvement, they are unsuitable as signals for informing the public about hospital differences in health outcomes.

**Supplementary Information:**

The online version contains supplementary material available at 10.1007/s10198-021-01333-w.

## Introduction

Quality measurement plays an important role in many health systems. Quality measures provide valuable insights for internal quality improvement projects, hospital management and external health authorities. These measures are also used in tools to compare providers and, as such, support patient choice for providers. More recently, quality measures have become a vital element of value-based payment, a new funding mechanism of health care that emphasizes a shift from volume towards value of care. Historically, measurement has mostly focused on hospital care and has used Donabedian’s framework of structure, process and outcome indicators [1, 2]. Given an empirically established structure–process–outcome relationship, the presence of good structures (e.g., the presence of an antibiotic protocol) and the presence good processes (e.g., administering antibiotics accordingly to the implemented protocol) increase the likelihood of achieving favorable outcomes of care (e.g., absence of a surgical-infection site) [3, 4]. Outcome indicators are considered as most important for health care consumers (patients), as they ultimately seek health care to improve their health, and, conditional the health outcome, are less interested in structures and processes used to produce it [5]. Structure and process indicators are generally considered to be highly informative for hospital management and internal quality improvement; these indicators provide more detailed and actionable information on how to improve the quality of care [6]. Therefore, sets of indicators consist ideally of all three types.

However, outcome indicators have been underrepresented in public reporting in the last decades. Pross and colleagues have analyzed hospital quality measures in five countries (England, Germany, the Netherlands, Sweden and the US). They conclude that “in most countries, measuring, reporting, and rewarding quality still focus on process indicators rather than on outcomes” and that “despite the relevance of outcome indicators and advances in risk adjustment, comprehensive reporting of risk-adjusted outcomes is scarce” [7]. The dominant use of process indicators has usually been justified by their many advantages relative to outcome indicators in terms of measurement; for example, necessary data are often already available in medical records, sophisticated case-mix adjustment and large sample sizes are generally not required [6].

The dominant use of process indicators has important implications in the context of public reporting. When users of public reported measures—such as patients—have to compare providers and select their preferred provider, they have to rely mainly on process indicators as proxies for outcome indicators. This also holds for other users, such as health care payers who selectively contract providers on behalf of their members, and governmental authorities who may monitor hospital quality levels over time. In theory, the dominant focus in public reporting on process indicators should not be harmful. That is because—under Donabedian’s framework—structure and process indicators can serve as valid proxies for outcome indicators, provided that the underlying structure–process–outcome relationship has been empirically established [1]. Indeed, numerous experimental and observational studies using individual-level data have demonstrated strong causal relationships and associations. These studies have provided a solid scientific base for current quality measures {see, for example, the specification of indicators measured by Centers for Medicare and Medicaid Services (CMS) [8]}. However, publicly reported quality measures are aggregated at hospital level. Few studies have investigated the structure–process–outcome relationship using hospital-level data. These observational studies have showed mixed results [9–14], often in conflict with those of experimental studies conducted previously.

### Observational research

Most of the studies based on publicly reported measures have been conducted in the US using CMS data and have focused (1) on heart conditions and pneumonia or (2) on surgical procedures. In the first group, two studies compared process indicators to case-mix adjusted mortality ratios reported for 2004. One study concluded that process indicators were “modestly” associated with mortality ratios: 31 of 39 associations (79.4%) were significant, but the process indicators only predicted small differences in mortality scores [9]. In contrast, the other study showed consistent relationships: all 3 associations (100.0%) were significant [10].

The three studies in the second group have compared surgical process indicators to various case-mix adjusted outcome indicators and their findings also contradict each other. Two cross-sectional studies used measures reported for 2005 and 2008, and reported mostly non-significant associations: 1 of 21 associations (4.7%) and 1 of 16 associations (6.3%), respectively, were significant [11, 12]. In contrast, a longitudinal study (2005–2010) concluded that most surgical process indicators were associated with surgical-site infection: 2 of 3 associations (66.7%) were significant [13].

Similar studies conducted outside the US are scarce: one study focused on structure or process indicators of hip replacement and surgical-site infection in Dutch hospitals (2008–2010). Their cross-sectional analyses revealed that only 1 of 18 associations (5.6%) was significant and correlated in the expected direction [14].

To sum up, the limited evidence has shown inconclusive findings. It is therefore unclear whether publicly reported structure and process indicators can serve as proxies for outcome indicators used to inform the public about the differences in health outcomes between hospitals.

We have identified two main reasons in the literature why the results of experimental studies may not correspond with those of observational studies based on aggregated data. First, the research questions differ in the groups of studies we compare. The experimental studies have been designed to answer whether a change in a particular structure or process of care would, on average, affect a patient’s health (i.e., efficacy) while holding everything else constant. The observational studies have been designed to inform patients on the average quality of care that depends on a complex interaction among a wide range of factors and actors in daily practice (i.e., effectiveness). While an indicator in an experimental study reflects the only change that could affect the outcome, it is one of many that could affect the outcome in an observational study. Whether the indicator is correlated with the outcome it aims to affect therefore depends on the correlation of that indicator with all the other relevant factors and actors. Because of this difference in the questions of both types of studies answer, they both may provide valid and reliable results, yet point in opposite directions.

Second, the conditions under which the indicators have been measured often differ (hereafter referred to as measurement conditions) and relate to reasons such as the lack of randomization in observational studies. Supplementary Material 1 provides a (non-exhaustive) overview of differences in measurement conditions between observational and experimental studies, and their potential effects on the study’s results. However, in the literature of health service research, the concept of aggregation bias is often overlooked according to Finney and colleagues as the studies use data at different aggregation levels [15]. More specifically, assuming that effects measured at the aggregated level of hospitals will translate into the same effects at the individual level of patients may lead to aggregation bias^1^ (also referred to as cross-level bias or the ecological fallacy). The concept of aggregation bias implies that the absence of consistent relationships at the aggregated hospital level does not refute the demonstrated causal effects at the individual patient level, nor the opposite.

### Our study

In this study, we aim to determine whether structure and process indicators provide useful signals for informing the public about the differences in health outcomes between hospitals. More specifically, we have assessed the extent to which structure and process indicators are correlated with outcome indicators in the context of compulsory public reporting in hospitals in the Netherlands. It is important to note that our aim is not to conduct an evaluation study of quality indicators based on observational data, nor to refute the findings of experimental studies. While most previous research has been conducted in the US hospital system, we have focused on the Dutch hospital system. Our research has allowed us (1) to include an important part of the Dutch hospital population and (2) to use recent data covering an extended time period. The latter has also allowed us to perform (3) a time-series assessment (4) on conditions not yet or seldom investigated in literature.

The US and Dutch health systems have similarities. First, both systems have relatively high healthcare expenditures: in 2015, the Dutch healthcare expenditure as share of gross domestic product was equal to 10.7%; in the US, this share was equal to 16.9%. Second, the two systems are characterized by provider competition to stimulate effective price and quality competition [17, 18]. Moreover, both systems rely mainly on process indicators to measure hospital quality [7]. Following the international trend, outcome indicators have been gradually included in Dutch indicator sets so that most current sets contain at least one outcome indicator.^2^ Nevertheless, the large majority of the outcome indicators are not adjusted for case-mix.^3^

The Dutch hospital system has some particularly relevant features for conducting this research. Most previous findings have been conducted on CMS data. Hence, they may not be generalizable to the total hospital population (all-payers). In addition, the accuracy and completeness of CMS data has often been debated [20]. In contrast, all Dutch patients have had universal access and nearly all Dutch hospitals have been legally obligated to publicly report quality measures; few hospitals have been exempted by the Dutch Healthcare Authority as, for example, they only provide out-patient care or refer patients to a nearby hospital for more complex hospital care [21]. As a consequence, the measures included reflect a significant part of total hospital population.

Additionally, Dutch quality measures have been developed with national government oversight and in close collaboration with scientists, providers, patient representatives and health insurers. Public reporting has been standardized by means of government guidelines and oversight, followed by national data quality check and case-mix correction. Final indicator values have been checked by the hospitals prior to publication [21]. Moreover, unlike CMS hospitals, Dutch hospitals have not been directly penalized with a reduction of annual fees if they have failed to report on quality measures [22]. This should reduce adverse behavioral effects.

Furthermore, the previous studies have generally been limited to cross-sectional analyses, and have often used data collected in the first few years of the quality measurement programs. Currently, Dutch clinical quality indicators have been implemented for over a decade and for a variety of medical conditions. This has allowed us to update findings of the previous study conducted on Dutch hospitals [14], and to perform a time-series assessment on conditions not yet included in previous research.

## Methods

Given that we aimed to study the structure–process–outcome relationship in the context of compulsory public reporting, we focused specifically on publicly reported measures that were accessible to anyone free of charge and were aimed to inform the public about the differences in clinical quality across hospitals. We selected hospital-generic and condition-specific measures. For the former, we included two in-hospital mortality indicators as measures to reflect the overall quality level of a given hospital: hospital standardized mortality ratio (HSMR) reported at hospital level and standardized mortality ratio (SMR) reported per related diagnosis group. For the latter, we selected indicator sets per medical condition.

Given the mandatory nature of reporting HSMR and SMR scores, we considered these measures to be highly relevant given our study context of public reporting. The Dutch government has made the reporting of HSMR and SMR mandatory despite research showing that higher case-mix adjusted mortality rates were not consistently associated with healthcare service with lower quality levels [23]. In accordance with Gaynor and Town who stated that “hospitals are thus not choosing mortality, but choosing a quality of service level that has an impact on mortality”, we used in-hospital mortality as generic measure to reflect the overall quality level of a given hospital [18]. Following Gaynor and Town’s line of reasoning, we assumed that HSMRs and SMRs incorporated factors known to affect quality of care such as hospital culture rather than the actual outcomes of the medical care provided. Given that it may take several years to change such a hospital culture, HSMRs and SMRs were thus expected to reflect the overall hospital quality level of past years. Given their broad scope (i.e., total hospital population), we expected that HSMR and SMR analyses may not result in consistent significant correlations. However, the direction of the relationship should correspond with the direction that was to be expected based on theory.

We focused on indicator sets that, besides structure and process indicators, contained one or more (case-mix adjusted) condition-specific outcome indicators (CSOs). CSOs reflected a more direct result of healthcare services relative to HSMRs and SMRs. Importantly, these indicator sets have served as input for online comparative tools {see, for example, [24, 25]}. Given the proximity of structure and process indicators to CSOs in the process of care provision, assessments based on CSOs should provide the strongest evidence relative to those based on HSMRs and SMRs, i.e., a larger share of strong significant relationships with their expected direction.

### Indicators

In the Netherlands, the National Bureau of Statistics computes HSMRs and SMRs, while hospitals are responsible for the compulsory publication of their scores. For these calculations, in-hospital mortality rates are grouped by diagnosis code. For each group, case-mix adjustment is performed after which the ratio of observed and expected number of patient deaths is multiplied by a 100 to obtain a SMR. The HSMR is then computed as the weighted overall score per hospital based on all SMRs [26]. Mortality rates are naturally negatively framed; low mortality scores imply fewer patient deaths, and lower scores on HSMRs and SMRs indicate higher quality levels, and vice versa. In our study, HSMRs (reported as annual scores) and SMRs (reported annually as 3 years pooled scores) reported for 2011–2018 were collected from the hospital’s own website and comparative websites [24, 25]. Table 1 provides a summary of all 24 included indicators (one HSMR, three SMRs, ten CSOs, three structure and seven process indicators), while a complete overview is provided in Supplementary Material 2.

**Table 1:** Overview of included indicators

Definition	Stratification	Indicator	Type	Level of evidence^c^	Year
Hospital patients					
Ratio of observed and expected number of in-hospital patient deaths (standardized at 100)	All diagnosis codes	HSMR	Outcome		2011–2018
	Breast cancer	SMR (breastca)	Outcome		2012–2018
	Colon cancer	SMR (colonca)	Outcome		2012–2018
	Rectum and anal cancer	SMR (rectumca)	Outcome		2012–2018
Breast cancer patients					
Share with an incomplete tumor resection^a^ after first breast conserving surgery	Invasive breast cancer	cso91	Outcome		2011–2018
	DCIS	cso92	Outcome		2011–2017
Number receiving surgical treatment		v10	Structure	1 [27]	2011–2018
Number receiving timely surgical treatment after diagnosis		v11	Process	4	2011–2017
Share receiving timely radiotherapy after chemotherapy		v12	Process	4	2014–2018
Colorectal cancer patients with surgical resection					
Share with an incomplete tumor resection^a^	Colon cancer	cso96	Outcome		2014–2018
	Rectum cancer	cso97	Outcome		2011–2018
Share who developed treatment-related complications (case-mix adjusted)	Colon cancer	cso91	Outcome		2014–2018
	Rectum cancer	cso92	Outcome		2014–2018
Share for which failure-to-rescue had occurred (case-mix adjusted)^b^		cso95	Outcome		2014–2018
Number receiving surgical treatment		v10	Structure	1 [28–30]	2011–2018
Share who received any form of treatment within 5 weeks after diagnosis	Colon cancer	v11	Process	4	2014–2018
	Rectum cancer	v12	Process	4	2014–2018
Hip replacement surgery patients					
Share who developed a deep surgical infection site post-operative	Within 6 weeks	cso91	Outcome		2011–2013
	Within 30 days	cso92	Outcome		2014–2014
	Within 90 days	cso93	Outcome		2015–2018
Number receiving orthopedic specialist surgical treatment		v10	Structure	1 [31, 32]	2011–2018
Share receiving timely antibiotic prophylaxis		v11	Process	1 [3, 4]	2011–2013
Share receiving peri-operative antibiotic prophylaxis		v12	Process	1 [3, 4]	2011–2013
Share with complete Dutch arthroplasty register information		v13	Process	4	2015–2018

*CSO* condition-specific outcome, *DCIS *ductal carcinoma in situ, *HSMR* hospital standardized mortality ratio, *SMR *standardized mortality ratio per related diagnosis group

^a^An incomplete tumor resection reflects a resection in which tumor cells have been observed in the circumferential resection margins (often referred to as an irradical resection). Irradical resection have been associated with unfavorable outcomes such as higher tumor recurrence rates [33]. Radical resection in which no tumor cells have observed in that margin are associated with favorable outcomes such as improved survival rate [34]

^b^The failure-to-rescue measure reflects the share of avoidable deaths. Hospitals with low rates are considered to be more successful in saving a patient’s life in the situation that surgical-related complications had occurred [35]

^c^Dutch scientific committees assess the content validity of a given measure using scientific evidence. Evidence is ranked according to the hierarchy of evidence while using the GRADE methodology. The strength of evidence ranges from the strongest level 1 to the weakest level 4

We obtained indicator sets (2011–2018) for three conditions from the National Health Care Institute database [36]. Similar to several previous studies described in the introduction section, we focused on a surgical procedure (hip replacement), but we also considered hospital quality for breast and colorectal cancer. The latter conditions were considered highly relevant as they ranked top three of all cancers in terms of the burden of disease in the Netherlands in 2015 [37].

Although the selected sets included several CSOs, we included those that had been measured for 4 years or longer (i.e., half of our study period) and were still included in the current set. As shown in Table 1, ten CSOs were included that reflected the following domains: (1) tumor‐positive resection margins (breast and colorectal cancer), (2) deep surgical infection site (hip replacement), and the case-mix adjusted measures for colorectal cancer, (3) treatment-related complications and, (4) failure to rescue. All CSOs were reported as percentages and, as with HSMRs and SMRs, were negatively framed.

As shown in Table 1, we selected three structure and seven process indicators that (1) had been measured for 4 years or longer and were still included in the current set, and (2) those that had content validity, irrespective of their measured time period. The former was relevant for our study as these indicators have been reported for multiple years to inform the public on hospital quality differences. The latter was relevant since, despite having face validity or being worthwhile measuring on their own, only part of the structure and process indicators have content validity, i.e., their underlying structure–process–outcome relationship has been supported by scientific evidence. Given their scientific base, the indicators with content validity were expected to have the strongest relationships with outcome indicators. To identify these indicators, we relied on the assessment of scientific committees described for each indicator in corresponding manuals and clinical guidelines. The included indicators reflected the domains of (patient or surgical) volume, timely start of treatment, complete registration of patient data and the administration of antibiotic prophylaxis. The structure indicators were measured on a continuous scale (e.g., the number of treated patients), while the process indicators were measured as percentages. All structure and process indicators were positively framed, i.e., higher scores implied a higher quality level, and vice versa.

### Statistical analyses

Our primary analyses were based on cross-sectional models as, in general, annual scores are presented to inform the public. We estimated univariate linear regression models for each outcome indicator per condition-year. These models assessed the extent to which structure and process indicators were correlated with outcome indicators in a given year. Subsequently, we estimated univariate regression models that took the longitudinal data structure into account, hereafter referred to as between-effects models. These models reduced the random variability in scores across years: average scores over the years for the dependent and independent variables were first computed and then included in the models. Furthermore, we estimated univariate random-effects and fixed-effects regression models that not only took the longitudinal structure of the data into account, but also considered the within-hospital variation. Unlike the cross-sectional and between-effects models, the random-effects and fixed-effects models reduced potential residual confounding as they captured unobserved time-invariant, between-hospital variation [38]. These models thus assessed the extent to which structure and process indicators were correlated with outcome indicators over time within a given hospital. Hausman's specification tests were performed to determine which specification was the most appropriate.

In each model, we looked at the significance level and the direction of the relationship. We expected inverse relationships: all structure and process indicators were positively framed, while all outcome indicators were negatively framed, thus foreshadowing a negative coefficient. If the observed and the expected direction of coefficients corresponded, the estimated relationship was labeled as ‘expected’; if otherwise, as ‘unexpected’. We performed subsequent trend analyses to test whether the obtained results systematically differed in terms of (1) the significance level and (2) the direction of the estimated relationship across main outcome indicator, condition and years. In the interest of brevity, trend analyses are described in more detail in Supplementary Material 3.

We stratified analyses by type of hospital. Academic hospitals may attract sicker patients than general hospitals due to their status [2], and may therefore be less likely to achieve high scores on outcome indicators despite closely following clinical guidelines. Moreover, scores on structure and process indicators may jointly affect a singular outcome indicator. We therefore repeated analyses using a multivariate approach: a single outcome indicator (dependent variable) and two or more structure and process indicators (independent variables). Furthermore, we conducted power calculations to assess potential statistical power issues. We expected to observe strong relationships indicated by a large coefficient of determination, often referred to as the explained variance or *R*-squared (*R*^2^). We assumed standardized betas equal to 0.7, 0.5 and 0.3 in our calculations which, respectively, explained 49%, 25% and 9% of the variance of the outcome indicator. The average cross-sectional sample consisted of 77.9 (standard deviation (SD)) 5.3 hospitals per year. The average longitudinal sample consisted of 79.7 (SD 5.4) hospitals per year with, on average, 5.1 (SD 5.1) measurements per hospital in total. We estimated the achieved power across the aforementioned effect sizes and sample sizes. To do so, we used the one-sample t-test (between-hospital effects) and repeated measures ANOVA (within-hospital effects), set the α-error probability at 5% and took the longitudinal data structure into account.

All models were bootstrapped (5000 bootstraps with replacement). Models and power calculations were estimated in *R* [39]. Results were considered statistically significant if *p* value < 0.05.

## Results

**Fig. 1:**
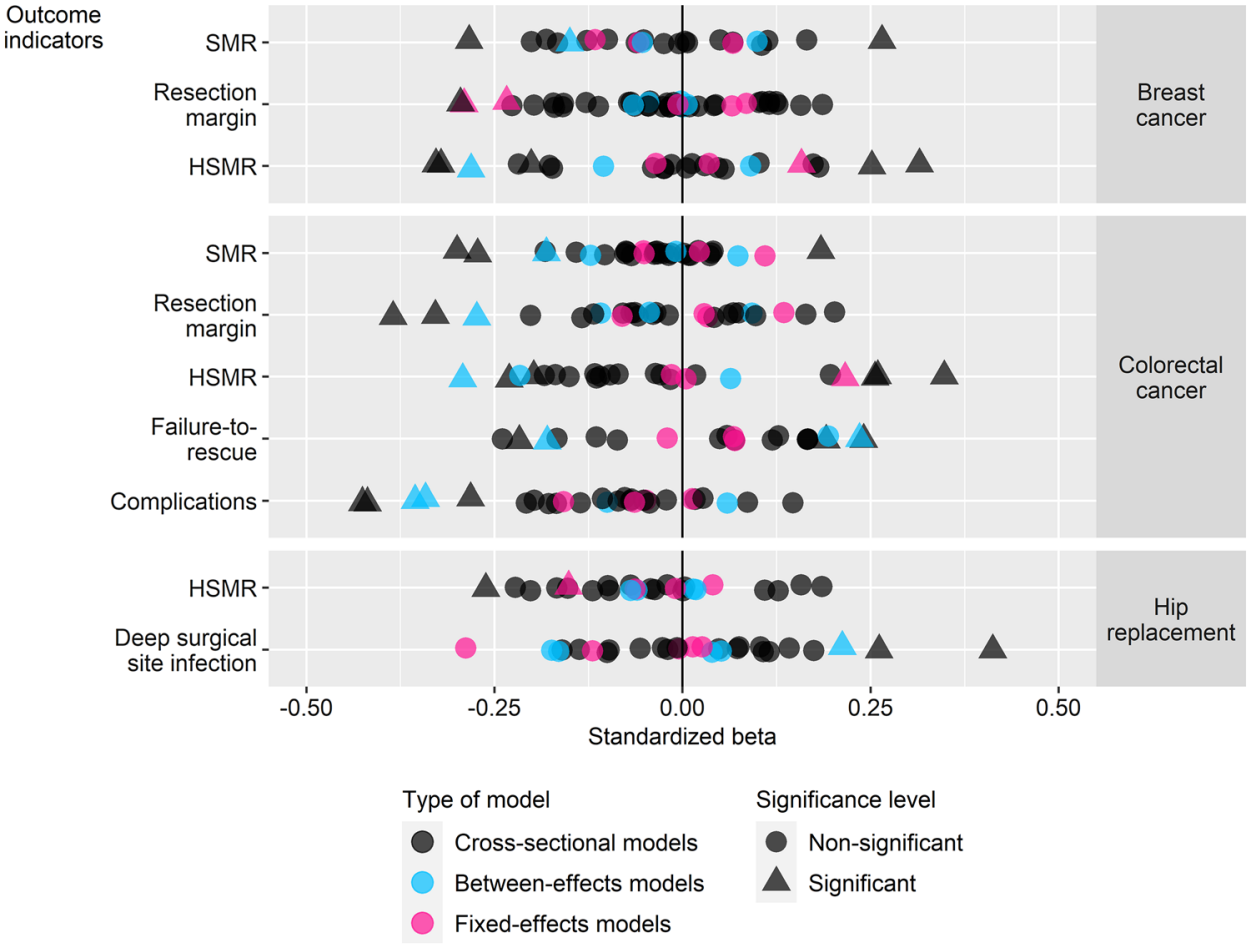
Standardized betas of main models stratified by type of model, condition and outcome indicator. A significant negative coefficient corresponds with evidence from literature and is labelled as ‘expected’. *HSMR* hospital standardized mortality ratio, *SMR* standardized mortality ratio per related diagnosis group

Figure 1 plots the standardized betas of the main models. In the interest of brevity, results of these models are summarized in Table 2 and provided in full detail (including corresponding trend analyses) in Supplementary Material 3. Table 3 shows the average magnitude of associations of the main models.

**Table 2:** Summary table: share of significant relationships between outcome indicators (dependent variable) and structure and process indicators (independent variable), stratified by type of model and condition

Model	Cross-sectional			Between-effects			Fixed-effects		
Relationship	Estimated	Significant		Estimated	Significant		Estimated	Significant	
Label	*n*	Expected (%)	Unexpected (%)	*n*	Expected (%)	Unexpected (%)	*n*	Expected (%)	Unexpected (%)
Breast cancer									
Overall	72	6.9	4.2	11	18.2	0.0	11	18.2	9.1
Stratified by outcome									
HSMR	20	15.0	10.0	3	33.3	0.0	3	0.0	33.3
SMR	18	5.6	5.6	3	33.3	0.0	3	0.0	0.0
CSO	34	2.9	0.0	5	0.0	0.0	5	40.0	0.0
Colorectal cancer									
Overall	96	10.4	6.3	18	33.3	5.6	18	0.0	5.6
Stratified by outcome									
HSMR	18	11.1	16.7	3	33.3	0.0	3	0.0	33.3
SMR	24	8.3	4.2	4	25.0	0.0	4	0.0	0.0
CSO	54	11.1	3.7	11	36.4	9.1	11	0.0	0.0
Hip replacement									
Overall	36	2.8	5.6	9	0.0	11.1	9	11.1	0.0
Stratified by outcome									
HSMR	18	5.6	0.0	4	0.0	0.0	4	25.0	0.0
CSO	18	0.0	11.1	5	0.0	20.0	5	0.0	0.0
All conditions									
Overall	204	7.8	5.4	38	21.1	5.3	38	7.9	5.3
Stratified by outcome									
HSMR	56	10.7	8.9	10	20.0	0.0	10	10.0	20.0
SMR	42	7.1	4.8	7	28.6	0.0	7	0.0	0.0
CSO	106	6.6	3.8	21	19.0	9.5	21	9.5	0.0
Stratified by year									
2011	14	14.3	0.0						
2012	18	0.0	0.0						
2013	18	0.0	5.6						
2014	29	10.3	6.9						
2015	31	9.7	3.2						
2016	33	3.0	6.1						
2017	33	9.1	6.1						
2018	28	14.3	10.7						

*CSO* condition-specific outcome, *HSMR* hospital standardized mortality ratio, *SMR* standardized mortality ratio per related diagnosis group

**Table 3:** Average magnitude of the observed relationships in main analyses, stratified by type of model

Model	Cross-sectional		Between-effects		Fixed-effects	
Observed direction	Expected	Unexpected	Expected	Unexpected	Expected	Unexpected
Label	Mean (SE)	Mean (SE)	Mean (SE)	Mean (SE)	Mean (SE)	Mean (SE)
All conditions						
All associations	− 0.093 (0.065)	0.086 (0.060)	− 0.089 (0.061)	0.062 (0.052)	− 0.070 (0.071)	0.051 (0.037)
Significant associations	− 0.297 (0.070)	0.271 (0.066)	− 0.257 (0.078)	0.224 (0.016)	− 0.225 (0.070)	0.187 (0.041)

*SE* standard error

In cross-sectional models (Fig. 1 and Table 2), no systematic association could be detected as the few significant relationships {across all conditions: 27 of 204 analyses (13.2%)} were scattered across conditions and outcome indicators, and observed for both expected and unexpected labels. Trend analyses confirmed the lack of systematic trends in significant relationships across (1) labels, (2) outcome indicators, (3) conditions and (4) years with two exceptions. Although fewer significant relationships were observed for unadjusted outcome measures (i.e., unadjusted CSO) relative to their adjusted counterparts (i.e., HSMR, SMR and adjusted CSO), the corresponding distribution of unexpected and expected labels did not differ. Additionally, no significant relationship was observed for any of the models for reporting year 2012. Furthermore, following Cohen’s recommendations^4^ for interpreting the magnitude of a correlation [40], the average magnitude of the associations (Table 3) was considered to be small in strength across all relationships (i.e., significant and non-significant) and moderate in strength across only significant relationships; for instance, a significant relationship labeled as expected had, on average, a standardized beta equal to − 0.297 (SD 0.070). More importantly and as supported by trend analyses, (5) similar results were found among indicators with the strongest strength of evidence (Supplementary Material 3): the few significant relationships (12 of 106 relationships, 11.4%) were equally distributed in terms of expected and unexpected labels (5.7% of all relationships, each), and were scattered across various outcome indicators and years.

In between-effects models (Fig. 1 and Table 2), a higher share of significant relationships was observed (10 of 38 analyses, 26.3%) on average and relative to the cross-sectional models. However, despite this higher share, the scattered distribution of significant relationships across (1) labels, (2) outcome indicators and (3) conditions persisted; the lack of any systematic trends was confirmed by trend analyses. In addition, all relationships observed as significant in between-effects models (*n* = 10) were not only found to be significant in corresponding cross-sectional models (i.e., one or more occasions), but also had the same direction (i.e., relationships were labeled as expected in both cross-sectional and between-effects models, and vice versa). Relative to the cross-sectional models, the average magnitude of the associations (Table 3) remained to be small in strength across all relationships and moderate in strength across only significant relationships. Furthermore, (4) similar results were observed among indicators with the strongest level of evidence (Supplementary Material 3): 4 of 19 analyses (26.3%) revealed significant relationships with a similar distribution of expected versus unexpected labels (15.8% and 5.3% of all relationships, respectively).

In the within-hospital comparison, the fixed-effects models (Fig. 1 and Table 2) produced similar findings to those of the between-hospital comparison. Few significant relationships were observed: 5 of 38 estimated models (13.2%). As confirmed by trend analyses, they lacked any systematic trend in terms of (1) the distribution of expected and unexpected labels, (2) outcome indicators and (3) conditions with the exception that no significant relationships were observed for models based on SMR. Similar to the cross-sectional and between-effects models, the average magnitude of the associations (Table 3) was also considered to be small in strength across all relationships and moderate in strength across only significant relationships. Moreover, Hausman tests indicated that a random-effects specification was more appropriate than a fixed-effects specification in 8 of 38 analyses (21.1%), on all occasions, the estimated relationship was not significant in either in the random-effects model, the fixed-effects model, or in both. Given the limited impact on the overall findings, results of the random-effects models and corresponding Hausman tests are only provided in Supplementary Material 3.

In additional analyses (Supplementary Material 3), the lack of systematic significant relationships persisted (1) in models stratified by type of hospital and (2) in models using multivariate approach, while (3) the power calculations revealed that, in general, the average sample had ample power to detect relevant effect sizes. In stratified analyses, models based on general hospitals revealed a significant relationship more frequently (31 of 204 analyses, 15.2%) than those based on academic hospitals (2 of 200 analyses, 1.0%). Similar to the findings of the main cross-sectional models, significant relationships lacked any systematic direction in terms expected or unexpected labels (general hospitals: 6.9% and 8.3% of the estimated relationships, respectively; academic hospitals: 0.5% for each label). On all occasions that a significant relationship was observed in both the stratified and the main model, the relationship had received the same label in both models.

In multivariate analyses and relative to the main cross-sectional models, results remained similar. A significant relationship was observed in 21 of 192 analyses (10.9%) with a relatively even share of expected (7.3%) and unexpected labels (3.6%). When the observed relationship was significant in both models, the assigned label corresponded.

Furthermore, the power of the average sample size was ample considering the conventional parameter, i.e., power > 80% [40]. For the between-hospital comparisons, the power was estimated at approximately 100.0% across all sample sizes for the expected standardized betas equal to 0.5 and 0.7, and considered to be borderline (i.e., 76.4–79.1%) across all sample sizes for the expected standardized beta equal to 0.3. For the within-hospital comparison, the estimated power approximated 100.0% across all tested effect sizes and sample sizes.

## Discussion

This study assesses—in the context of compulsory public reporting—the extent to which structure and process indicators correlate to outcome indicators in Dutch hospitals. More specifically, we have determined whether structure and process indicators can be used as signals to inform the public about the differences in health outcomes between hospitals. Our research contributes new evidence to literature and has allowed us to corroborate previous findings that appear to have had limited impact on policy makers [7].

### Principal findings and explanations

Findings from our primary (cross-sectional) models have shown that structure and process indicators aggregated at hospital level are not correlated with outcome indicators. Across conditions, outcome indicators and years, we have observed few significant relationships (13.2%) with no trend in terms of the observed direction. More importantly, we have not been able to detect consistent relationships among indicators with the strongest strength of evidence. Our findings are consistent with those of several observational studies based on publicly reported hospital-level data [9–14]. However, our findings conflict with those of experimental studies using individual-level data, these studies have shown that the presences of structures and processes of health care have a strong and causal effect on health outcomes.

Literature provides two main explanations for these differences: (1) the differences in the research questions and (2) the validity and reliability of studies. The former allows both groups of studies to be both valid and reliable, even if they provide seemingly contradictory results. The experimental studies have focused on the effect of the structures or processes of health care on health outcomes at patient level. In contrast, the observational studies have focused on the correlation between structure and process indicators at hospital level on the one hand and outcome indicators on the other hand. The correlation at hospital level can be affected by factors other than the ones affecting the causal effect of the structures and processes on health outcomes at patient level.

With respect to the validity and reliability of studies, explanations can be divided into behavioral and methodological explanations. From a behavioral perspective, behavioral effects such as gaming come into play in the practice of public reporting [2, 41]. Favorable scores on quality measures may have financial benefits for hospitals and individual providers and managers, such as career promotions [2]. Conversely, unfavorable scores may lead to financial losses, such as departments being shut down due to national inquiries and loss of jobs [42, 43]. Artificially boosted scores on indicators may thus prevent the detection of significant relationships.

From a methodological perspective, methodological explanations can be further divided into two groups. The first subgroup captures the concept of aggregation bias. One might ask why some hospitals with better procedures—that have been shown to improve outcomes at patient level—do not perform better on measures reported at hospital level. Suppose that a given hospital performs poorly on the outcome indicator ‘surgical infection rate’. Physicians in that hospital may react to the high rate by improving factors that they can control, such as the administration of antibiotics. The physicians may therefore focus on carefully following guidelines by administering broad-spectrum antibiotics as indicated during hip surgery. Consequently, causal mechanisms may be reversed as unfavorable scores on outcome indicators affect scores on process indicators. The concept of aggregation bias underlines the view that, if hospital-level results show no systematic correlation, they should not be interpreted as a demonstration that no causal effects exist at the patient level, nor the opposite.

The second subgroup of methodological explanations might explain the differences between both types of studies. Under the assumption that the same causal effects exist both at hospital level and at patient level (i.e., aggregation bias does not play a role), any divergence between causal effects and correlations can be due to confounding, selection bias, measurement bias and reliability issues. Selection bias is less likely to affect the results as—in principle—all patients who have received hospital treatment and met the indicator’s inclusion criteria have been included in our analyses. More likely causes for bias are systematic differences in reporting and unobserved confounding, in particular for the outcome indicators in the observational studies.

To assess the issue of confounding and reliability, we have performed additional analyses. With respect to confounding, we have stratified our cross-sectional models by type of hospital as an academic status of a hospital may affect our results. It is reasonable to assume that due to their teaching activities, academic hospitals have a stronger focus on scientific insights and guidelines than general hospitals. Academic hospitals therefore may implement these guidelines sooner and more effectively than others. However, their outcomes might ultimately suffer from the lack of experience of junior physicians or continuously changing teams. The stratified analyses have revealed a very small share of significant relationships for academic hospitals (1.0%) relative to general hospitals (15.2%). This finding may likely be attributed to the small number of Dutch academic hospitals (between 2011 and 2018: *n* = 8) relative to that of general hospitals (between 2011 and 2018: *n* = 71–84). Nevertheless, the share of significant relationships for only general hospitals remains small with no consistent direction detected in terms of expected and unexpected labeled relationships. We therefore conclude that the presence or absence of an academic status of a hospital does not explain the lack of systematic correlations observed in our main analyses.

In addition, we have conducted multivariate analyses, because scores on structure and process indicators may jointly affect a singular outcome indicator. We have found findings similar to those of univariate models and our overall conclusion therefore holds.

Moreover, our power calculations have showed that the average cross-sectional samples may lack the statistical power to detect specific effect sizes. Therefore, the issue of reliability can explain why some correlations are not significant. We have performed between-effects models to improve power as these models use the longitudinal data structure to reduce the effects of random variability in scores over the years. Although we observe slightly higher shares of significant relationships (26.3%), the large majority of the estimated relationships remain non-significant. Given that the between-effects models support our cross-sectional findings, we therefore conclude that the lack of systematic correlations cannot be attributed to reliability issues.

To evaluate the effect of both public reporting and case-mix differences, we have investigated the structure–process–outcome relationship in a within-hospital comparison using fixed-effects models. These models have also revealed small shares of significant relationships (13.2%) with no systematic direction in terms of expected and unexpected labels. These findings add a new dimension to the aforementioned conclusion (i.e., structure and process indicators aggregated at hospital level are not correlated with outcome indicators): changes in publicly reported structure and process indicators are not correlated with changes in related outcome indicators within a given hospital over time. We therefore conclude that unobserved case-mix differences and systematic reporting differences do not explain the lack of systematic correlation.

### Implications

Our findings have important implications for policy and practice. While most quality indicators may have initially been developed with sole aim to improve internal hospital quality, these measures are currently reported publicly and serve multiple goals: (1) to support accountability to health authorities, (2) to allow (international) comparisons for health policy, (3) to facilitate patient choice and (4) the selective contracting of providers in health care procurement. The results of our between-hospital analyses indicate that, in the context of public reporting, structure and process indicators will not help those who use these measures—such as patients, physicians and health care procurers—to identify hospitals with the better outcomes of health care. The results of our within-hospital analyses imply that these indicators are also unsuitable as proxies for outcome indicators to monitor changes in health outcomes over time within hospitals. Although publicly reported structure and process indicators may still inform users of these indicators about the presence of practice variation in itself, users should be aware that any observed variations do not appear to translate into differences in the outcomes of health care. While still valuable for internal hospital management, structure and process indicators are unsuitable as proxies for outcome indicators and should therefore not be used as signals to inform the public on differences in health outcomes between hospitals. Additional efforts that push the implementation and public reporting of outcome indicators are therefore recommended.

### Strengths and limitations

A strength of our study is that we have generated new evidence: whereas most previous research has focused on the US based on CMS data [9–14], we have conducted the assessment in the Dutch hospital system. We also add unique evidence to literature as we have focused on hospital quality for cancer; conditions that have been limited studied in previous research. More importantly, our findings corroborate those of several previous studies conducted in different settings and in a different health system. Our overall conclusion seems to hold across different conditions and across different health systems which provide credence to the generalizability of our findings. Another study strength is that we have been able to address several limitations of previous studies in two ways. (1) Most observational studies relying on publicly reported data have been limited by cross-sectional data. Our data (2011–2018) has enabled us to enrich these cross-sectional analyses with longitudinal analyses that, in turn, allows us to correct for unobserved time-invariant confounding factors, as well as to study trends over time. (2) As most previous findings are based on CMS data, they may therefore have suffered from selection bias as these data may not be representative to certain patient groups, e.g., the less vulnerable. Our findings, however, are based on a large part of the hospital population as nearly all Dutch hospitals are legally obligated to publicly report on quality measures and all Dutch patients have universal access to hospital care.

We also need to acknowledge certain study limitations. First, we have been unable to include patient-reported outcome measures (PROMs) and patient-reported experience measures (PREMs) in our assessment. These measures have either not been reported (breast cancer and colorectal cancer) or have only recently been included in the set (hip replacement).^5^ Second, we have assumed that if outcome indicators on the one hand and related structure and process indicators on the other hand, are not correlated, that outcome indicators are more valid indicators of actual outcomes than structure and process indicators [5]. While we cannot completely rule out the possibility that the opposite is true, the observed lack of consistent relationships still remains problematic.

## Conclusion

It is often well established that improving procedures are associated with improved health outcomes. However, our results have indicated that hospitals that compare well in terms of publicly reported structure and process indicators do not consistently outperform other hospitals in terms of corresponding publicly reported outcome indicators. Moreover, our study has indicated that even within hospitals, changes in structure and process performance measured with hospital-level indicators do not correlate with changes in outcomes they aim to improve, as measured with outcome indicators. The lack of significant relationships has also been observed among indicators with the strongest underlying study designs and has persisted in stratified analyses by type of hospital and in multivariate analyses. We therefore conclude that while still valuable for internal hospital management, structure and process indicators are generally unsuitable as proxies for outcome indicators. They are therefore unsuitable as signals for informing the public on differences in health outcomes between hospitals.

## Supplementary Information

Below is the link to the electronic supplementary material.

Supplementary file1 (DOCX 27 KB)

Supplementary file2 (XLSX 33 KB)

Supplementary file3 (XLSX 139 KB)

## Data Availability

The data that support the findings of this study are available from the website of The Dutch National Health Care Institute [36], the hospital's own website and comparative websites [24, 25].

## Footnotes

1 Aggregation bias arises from the misconception that the characteristics of an individual are equal to the average characteristics of the group, thereby ignoring the variability (distribution) in these characteristics [16].

2 Based on the authors’ investigation of the indicator manuals [19]. For 2018, 43 sets were reported: 18 sets (41.9%) did not include any outcome indicator, 1 set (2.3%) included only patient-reported outcome indicators, 16 sets (37.2%) included only clinical reported outcome indicators, while 8 sets (18.6%) contained both types of indicators.

3 Among the 24 sets that included clinical reported outcome indicators, only 7 sets reported that some form of case-mix correction had been performed, e.g., stratification by age strata or standardization.

4 A standardized beta equal to 0.10, 0.30 and 0.50 were considered to be small, medium and large in magnitude, respectively.

5 Several PROMs have been reported on voluntary basis in 2016. Since 2017, the reporting on these measures has been made compulsory.
