# Supplementary material for: On the correlation between outcome indicators and the structure and process indicators used to proxy them in public health care reporting
Source: Eur J Health Econ. 2021 Jun 30;22(8):1239–51. doi: 10.1007/s10198-021-01333-w (PMC8526472; doi:10.1007/s10198-021-01333-w)
Supplement: Supplementary file 1 — Supplementary file1 (DOCX 27 KB) [file 10198_2021_1333_MOESM1_ESM.docx]

Supplementary Material 2

*Manuscript: On the correlation between outcome indicators and the structure and process indicators used to proxy them in public health care reporting.*

*Authors:* B.H. Salampessy, F.R.M. Portrait, E. van der Hijden, A. Klink and X. Koolman.

*Table S1. A (non-exhaustive) overview of possible differences in measurement conditions between studies and of their potential effects on study results.*

| Factor | Difference in measurement conditions between studies | Potential effects on study results |
| --- | --- | --- |
| **Methodological perspective** | | |
| Randomization or the lack thereof. | Unlike experimental studies, patients are not randomly assigned to hospitals in practice. | Given that patients’ characteristics are likely to differ systematically across hospitals, observational studies may be more prone to residual confounding [1,2]. |
| Use of different data-levels. | Experimental studies generally use patient-level data, whereas observational studies use aggregated hospital-level measures [3]. | Comparisons of study findings measured at different data-levels may result in aggregation bias (also referred to as the ecological fallacy): the misconception that the characteristics of an individual are equal to the average characteristics of the group, thereby ignoring the variability (distribution) in these characteristics [4]. |
| Strict inclusion criteria, or the lack thereof. | Experimental studies generally apply strict inclusion criteria for participants, e.g., no co-morbidities [1]. Hospital care is provided to a wide range of patients, i.e., less homogeneous. | This difference in inclusion criteria may limit the generalizability of findings of experimental studies to the total hospital population. |
| Different type of measurement errors. | Experimental studies generally have extensive data quality control management in place to minimize measurement errors. In contrast, hospital data is known to be more prone to dependent errors, e.g., underreported co-morbidities [6], learning effects in the initial years of measurement [7], and following changes in the specifications of indicators. | Given that measurement errors in hospital data are unlikely to be independent and non-differential in nature, measurement bias may arise in a way that the observed relationships may be biased towards or away from the true relationships and thus biased in an undetermined direction. |
| **Behavioral perspective** | | |
| Possibility to game reported scores | Unlike experimental studies that have fixed inclusion criteria and a fixed study protocol for each study arm, hospitals may influence the treated patient population and, given ceteris paribus, may treat patients differently. | Hospitals may select patients in relatively good health and refuse to treat those in relatively poor health in order to increase the likelihood on a positive health outcome among the treated population. This may ultimately improve the hospital’s average score on the indicator [8]. |
| Possibility to multitask reported scores | Experimental studies generally aim to report the measured effects as broad and as complete as possible, e.g., include a wide range of measures, triangulation by conducting additional qualitative research. In contrast, hospitals are known to use the multidimensional nature of quality of care by focusing on the reported dimensions of quality, while falling short on the non-reported dimensions [9,10]. | Multitasking causes scores on quality measures to be conditional on whether these measures are publicly. |

# References

1. Hernán, M., Robins, J.: Causal Inference. Chapman & Hall/CRC, forthcoming, Boca Raton (2018)

2. Parast, L., Doyle, B., Damberg, C.L., Shetty, K., Ganz, D.A., Wenger, N.S., Shekelle, P.G.: Challenges in Assessing the Process–Outcome Link in Practice. J Gen Intern Med **30**(3), 359-364 (2015). doi:10.1007/s11606-014-3150-0

3. Finney, J.W., Humphreys, K., Kivlahan, D.R., Harris, A.H.S.: Excellent Patient Care Processes in Poor Hospitals? Why Hospital-Level and Patient-Level Care Quality-Outcome Relationships Can Differ. J Gen Intern Med **31**(Suppl 1), 74-77 (2016). doi:10.1007/s11606-015-3564-3

4. Robinson, W.S.: Ecological Correlations and the Behavior of Individuals. Am Sociol Rev **15**(3), 351-357 (1950). doi:10.2307/2087176

5. Stevens, A., Abrams, K., Brazier, J., Fitzpatrick, R., Lilford, R.: The advanced handbook of methods in evidence based healthcare. Sage, London (2001)

6. Burns, E.M., Rigby, E., Mamidanna, R., Bottle, A., Aylin, P., Ziprin, P., Faiz, O.D.: Systematic review of discharge coding accuracy. J Public Health (Oxf) **34**(1), 138-148 (2011). doi:10.1093/pubmed/fdr054

7. Mabry, C.D.: “Say it ain't so, joe”: Comment on “hospital process compliance and surgical outcomes in medicare beneficiaries”. Arch Surg **145**(10), 1004-1005 (2010). doi:10.1001/archsurg.2010.189

8. Rosenthal, M.B., Frank, R.G.: What Is the Empirical Basis for Paying for Quality in Health Care? Med Care Res Rev **63**(2), 135-157 (2006). doi:10.1177/1077558705285291

9. Campbell, S.M., Roland, M.O., Buetow, S.A.: Defining quality of care. Soc Sci Med **51**(11), 1611-1625 (2000). doi:10.1016/S0277-9536(00)00057-5

10. Dranove, D.: Chapter Ten - Health Care Markets, Regulators, and Certifiers. In: Pauly, M.V., McGuire, T.G., Barros, P.P. (eds.) Handbook of Health Economics, vol. 2. pp. 639-690. Elsevier, Amsterdam (2011)
